# Caracterización de las lesiones personales en el sistema estomatognático valoradas en el Instituto Nacional de Medina Legal y Ciencias Forenses, Regional Suroccidente, entre 2015 y 2020

**DOI:** 10.7705/biomedica.6102

**Published:** 2022-03-01

**Authors:** Lina-Alexandra Castañeda, María-Paula Quintero, Sandra-Milena Moreno-Correa, Freddy Moreno-Gómez, Ruby-Amparo Vázquez-Escobar

**Affiliations:** 1 Escuela de Odontología, Universidad del Valle, Cali, Colombia Universidad del Valle Universidad del Valle Cali Colombia; 2 Facultad de Ciencias de la Salud, Pontificia Universidad Javeriana, Cali, Colombia Pontificia Universidad Javeriana Pontificia Universidad Javeriana Cali Colombia; 3 Instituto Nacional de Medicina Legal y Ciencias Forenses, Regional Suroccidente, Cali, Colombia Instituto Nacional de Medicina Legal y Ciencias Forenses Cali Colombia

**Keywords:** violencia, ciencias forenses, odontología forense, heridas y traumatismos, sistema estomatognático, Violence, forensic science, forensic dentistry, wounds and injuries, stomatognathic system

## Abstract

**Introducción.:**

El dictamen de lesiones personales hace precisiones sobre el daño ocurrido en el cuerpo o en la salud de una persona. La valoración del odontólogo es de gran importancia en los casos en que se ha visto afectado el sistema estomatognático.

**Objetivo.:**

Caracterizar las lesiones personales que afectaron el sistema estomatognático como producto de actos violentos en 266 casos valorados en el Instituto Nacional de Medina Legal y Ciencias Forenses, Regional Suroccidente, entre 2015 y 2020.

**Materiales y métodos.:**

Se hizo un estudio descriptivo de corte transversal de las lesiones personales que afectaron el sistema estomatognático. Se incluyó información del dictamen de lesiones personales obtenida de la plataforma del Sistema de Información Forense de Clínica de Colombia (SICLICO) y asociada con la naturaleza de la lesión, las secuelas y la incapacidad médico-legal.

**Resultados.:**

Se observó una mayor frecuencia de agresores y de víctimas masculinas (94,4 y 64,3 %, respectivamente), especialmente en el grupo etario entre los 21 y los 30 años (32,6 %). Los contextos más frecuentes fueron los de violencia interpersonal (76,3 %) y violencia de pareja (19,9 %). El principal mecanismo de agresión fue el contundente (88,0 %). Las lesiones reportadas en tejidos blandos (83,5 %) afectaron tejidos periodontales (48,9 %) y labios (28,6 %) con edema (32,7 %) y heridas (22,9 %), en tanto que, en tejidos duros (55,1 %), afectaron los dientes (41,4 %) por fractura complicada en tercio cervical (19,2 %) y avulsión (18 %). Predominó la incapacidad definitiva (64,7 %), seguida de la de 20 días (28,6 %) y las secuelas funcionales (24,1 %).

**Conclusiones:**

Los tejidos periodontales y los dientes fueron las estructuras más afectadas, lo que ocasionó incapacidades considerables, y dejó secuelas funcionales y estéticas principalmente entre los hombres en edad productiva.

## Abstract (en)

**Introduction:**

Personal injury forensic reports diagnose and define the damage to the body and health of a person including the stomatognathic system by injuries produced as the consequence of other people's actions.

**Objective:**

To characterize the personal injuries affecting the stomatognathic system as a result of violent actions in 266 cases evaluated at the Instituto Nacional de Medina legal y Ciencias Forenses-Regional Suroccidente (Cali, Colombia) between 2015 and 2020.

**Materials and methods:**

We conducted a descriptive cross-sectional study to characterize the personal injuries affecting the stomatognathic system. The information was taken from the personal injuries forensic reports registered on the Colombian forensic registration system (Sistema de Información Forense de Clínica de Colombia, SICLICO) platform and associated with the nature of the injury including its consequences and the medico-legal incapacity issued.

**Results:**

We observed a higher frequency of male aggressors (94.4%) and male victims (64.3%), especially in the age group between 21 and 30 years (32.6%). The most frequent contexts were interpersonal violence (76.3%) and intimate partner violence (19.9%). The main aggression mechanism was blunt trauma (88.0%). The lesions reported in soft tissues (83.5%) affected periodontal tissues (48.9%) and lips (28.6%) due to edema (32.7%) and wounds (22.9%) while in hard tissues (55.1%), they affected the teeth (41.4%) due to complicated fractures in the cervical third (19.2%) and avulsion (18%). Definitive disability predominated (64.7%) followed by 20-day disability (28.6%) and functional sequelae (24.1%).

**Conclusions:**

Periodontal tissues and teeth were the most affected structures causing considerable disabilities, as well as functional and aesthetic sequelae that mainly affected men in productive age.

La odontología forense se define como la especialidad de las ciencias forenses encargada de brindar información técnica y científica a la justicia referente a los dientes y las estructuras que constituyen el sistema estomatognático, así como sobre traumas, enfermedades y tratamientos odontológicos, valorados por profesionales de salud dental, bucal y maxilofacial.

Se ciñe a lo estipulado en la legislación vigente -entre otros, el Código de Ética del Odontólogo Colombiano, Ley 35 de 1989 y la Ley 38 de 1993, por la cual se unifica el sistema de dactiloscopia y se adopta la carta dental para fines de identificación-, para los casos de identificación de seres humanos vivos o fallecidos y dictámenes especiales en el marco de desastres, exhumaciones, casos de responsabilidad profesional, maltrato infantil, abuso sexual y lesiones personales ^1^. Para ello, en el 2011, el Instituto Nacional de Medicina Legal y Ciencias Forenses (INML y CF) desarrolló una guía práctica para el examen odontológico forense a cargo de odontólogos y médicos funcionarios del Instituto y profesionales vinculados a los servicios de salud públicos y privados en todo el territorio colombiano, encargados de las pesquisas judiciales que involucren la evaluación del sistema estomatognático ^2^.

Las lesiones personales se definen como los daños (entendidos como toda alteración de la integridad física o mental de un ser humano) causados de manera voluntaria o involuntaria, y que presentan signos y síntomas generales, particulares y específicos ^3^.

En términos médico-legales, las lesiones personales corresponden a la alteración de la morfología o la función de estructuras, órganos, segmentos corporales y sistemas que, producidas por un agente traumático operado por un tercero o un agente externo, trastornan la salud y causan desequilibrios de mayor o menor gravedad ^4^. Por lo tanto, los peritos médicos y odontólogos que investigan y dictaminan sobre las lesiones personales en personas vivas, deben valorar las variaciones que presenta el sistema estomatognático para obtener información que pueda orientar sobre el tiempo transcurrido desde la lesión, la relación con algún objeto específico causante, y el lugar, las circunstancias y la secuencia de los hechos. Para ello, es necesario describir y localizar las lesiones morfológicas y funcionales a partir de un diagnóstico diferencial que responda a los interrogantes de las autoridades competentes en el dictamen de lesiones personales. Cuanto más pronto se haga este diagnóstico, mayor será su exactitud. Una vez determinado el daño corporal y de la salud, se deben estimar la gravedad de las lesiones, el tiempo de reparación biológica primaria expresado en días (incapacidad y secuelas médico-legales), las implicaciones penales (tipificación del delito, medida de aseguramiento y punibilidad) y el costo patrimonial (indemnización) ^3^.

Este reconocimiento médico-legal de las lesiones personales se realiza en casos de lesiones leves que no ponen en riesgo la integridad de la persona y no generan compromiso funcional o estético, o cuando haya lesiones reparadas con cicatrices que, conjuntamente con la lectura e interpretación de la historia clínica y las ayudas diagnósticas, evidencian su origen y curso, o en casos de lesiones graves que permiten al perito determinar claramente la incapacidad y las secuelas. Así, al iniciarse la investigación por el delito de lesiones personales, la autoridad competente ordena el reconocimiento médico-legal del lesionado para determinar: 1) la naturaleza de las lesiones; 2) el instrumento con que fueron causadas; 3) la incapacidad médico-legal, y 4) las secuelas médico-legales por deformidad física o perturbación funcional ^5^^,^^6^.

El dictamen odontológico de lesiones personales se requiere en casos de accidentes, mala práctica odontológica o violencia física, en los cuales se involucre alguna de las estructuras que conforman el sistema estomatognático, se haga necesario expedirse una incapacidad de restitución de forma y función, o se determine que existe una secuela que se extiende más allá del tiempo de la incapacidad, por daño importante en la morfología o función ^2^^,^^7^.

Por otro lado, dado que las lesiones personales suponen el uso de la fuerza en una situación de conflicto, la violencia, efectiva (aplicada) o virtual (amenaza), se encuentra en el centro y constituye uno de los factores determinantes de su resultado jerarquizado según el contexto sociocultural. Sin embargo, pese a la heterogeneidad y las múltiples causas de las lesiones personales y la transversalidad contextual de los diversos factores asociados, las intervenciones se han enfocado en la violencia intrapersonal, de tal manera que se ha priorizado la atención de algunos grupos poblacionales y las acciones se han concentrado en la determinación de patrones espaciales o temporales de criminalidad ^8^.

Si bien la violencia, como toda conducta humana, tiene raíces en la biología (el ser humano es agresivo por naturaleza), puede verse modificada mediante dispositivos socioculturales (el ser humano es pacífico o violento por cultura). En dicho contexto, entonces, la agresividad está en la base de la intención de causar daño en el otro. La violencia carece de función vital y de supervivencia (no es útil en el proceso evolutivo de selección o adaptación del ser humano), por el contrario, se activa en favor de ideas, creencias, prejuicios y valores apropiados a lo largo de la historia personal y la socialización. De allí que la intervención en el proceso de socialización sea determinante para reducir o potenciar la violencia, toda vez que es el propio ser humano quien dota a sus acciones agresivas de intencionalidad. La violencia, entonces, es un tipo de interacción social en la que se causa daño de manera intencional ^9^.

En las actuales condiciones culturales, sociales y económicas de nuestro país, los comportamientos y conductas irracionales de algunos, pueden venir acompañadas de agresiones físicas en los contextos de violencia común, violencia intrafamiliar y maltrato infantil, casos en que las lesiones personales involucran el sistema estomatognático, siendo los tejidos blandos periorales y los dientes las estructuras más vulnerables ^3^^,^^5^.

En este contexto, el objetivo del estudio consistió en caracterizar las lesiones personales por actos violentos que afectaron el sistema estomatognático en 266 casos valorados en el Instituto Nacional de Medicina Legal y Ciencias Forenses, Regional Suroccidente, entre el 2015 y el 2020 y registrados en la plataforma de SICLICO, y determinar los aspectos sociodemográficos del agresor y de la víctima, las características de los hechos, la incapacidad médico-legal y las secuelas morfológicas y funcionales.

## Materiales y métodos

Se hizo un estudio descriptivo de corte transversal de caracterización de las lesiones personales que afectaron el sistema estomatognático en 266 casos valorados por odontólogos forenses del Instituto Nacional de Medicina Legal y Ciencias Forenses, Regional Suroccidente, entre el 2015 y 2020. Se incluyeron todos los casos de lesiones personales ocasionados por violencia y se excluyeron aquellos de lesiones causadas por accidentes de tránsito y mala práctica profesional.

Según el Artículo 11 de la Resolución 8430 de 1993 del Ministerio de la Protección Social, y los principios éticos para las investigaciones médicas en seres humanos establecidos por la Asociación Médica Mundial en la Declaración de Helsinki, el riesgo de este estudio fue menor que el mínimo, pues no involucró a seres humanos de forma directa; sin embargo, dado que se manejó información de una base de datos institucional oficial en custodia, se requirió del aval del Comité Institucional de Revisión de Ética Humana de la Facultad de Salud de la Universidad del Valle y del Instituto Nacional de Medicina Legal y Ciencias Forenses para garantizar la protección de la identidad de los individuos y el manejo de la información con absoluta reserva.

Obtenidos dichos avales, se revisaron los resultados de los informes periciales de clínica forense depositados en el SICLICO para determinar los casos atendidos por odontología forense en el Instituto Nacional de Medicina Legal y Ciencias Forenses, Regional Suroccidente, entre el primer trimestre del 2015 y el primero del 2020. Debe señalarse que dicha herramienta recolecta la información en tiempo real para ampliar el estudio de la violencia en Colombia mediante las variables del sistema y según los estándares establecidos por el Instituto como órgano de referencia de la actividad pericial forense en Colombia.

Se obtuvo información preliminar de la edad referida, el relato de los hechos, la atención en salud, el examen médico-legal, el análisis, la interpretación y las conclusiones. Posteriormente, se organizó la información en una hoja electrónica de Excel®, utilizando numeración consecutiva para identificar cada caso y las categorías de estudio establecidas a partir de la Guía Práctica para el Examen Odontológico Forense, versión 03 ^2^, y el Reglamento Técnico para el Abordaje Integral de Lesiones Personales, versión 01 ^1^.

Las categorías tenidas en cuenta fueron: datos sociodemográficos de la víctima (sexo, edad, escolaridad, ocupación, etnia, lugar de nacimiento, orientación sexual, factor de vulnerabilidad), de los hechos (mes, hora, lugar -barrio y comuna-, contexto), del agresor (filiación con la víctima, sexo), de los agentes y mecanismos traumáticos, de los tejidos y estructuras afectadas (tejidos duros, tejidos blandos, dientes), del tipo de lesión, del tipo y carácter de las secuelas, y del tipo y número de días de la incapacidad.

Una vez se completó la información en la hoja electrónica, los datos obtenidos se procesaron en el *software* SPSS™, versión 21.0, mediante análisis cuantitativo univariado (estadística descriptiva).

## Resultados

En el total de 1.872 informes periciales de clínica forense revisados, se encontraron 268 casos de lesiones personales que afectaron componentes morfofuncionales del sistema estomatognático como producto de actos violentos. No se contaba con la información completa de dos de los casos, de manera que la muestra correspondió finalmente a 266 casos.

En cuanto a los datos sociodemográficos de la víctima, hubo más casos de lesiones personales en hombres (64,3 %) que en mujeres (35,7 %), siendo más frecuentes en individuos mayores de 18 años (77,9 %). La mayoría de las víctimas tenía algún grado de escolaridad, más frecuentemente la educación básica secundaria completa (41,6 %), y el 92,5 % refirió tener algún tipo de ocupación. La mayoría de las víctimas reportó no pertenecer a grupos étnicos (79,3 %), lo que los clasifica como mestizos caucasoides, y haber nacido en el Valle del Cauca (75,6 %). Según el Departamento Administrativo Nacional de Estadística (DANE), la categoría de etnia se adoptó a partir del Censo Poblacional del 2005 para referirse a los individuos que se reconocen como integrantes de los grupos étnicos indígena, afrocolombiano (incluye afrodescendientes, negros, mulatos, palenqueros de San Basilio), raizales del archipiélago de San Andrés y Providencia, y rom o gitano. En cuanto a la orientación sexual, una mayor proporción de víctimas se presentó como heterosexual (94,7 %) y no presentaban ningún factor de vulnerabilidad en el momento de los hechos (73,7 %); sin embargo, llama la atención que algunas de ellas se encontraban bajo los efectos de sustancias psicoactivas (16,2 %) (cuadro 1).

**Cuadro 1:** Datos sociodemográficos de la víctima

Sexo	n	%
Mujer	95	35,7
Hombre	171	64,3
**Grupo etario**	**n**	**%**
9-12	2	0,8
14-17	11	4,1
18-20	26	9,7
21-30	87	32,6
31-40	59	22,5
41-50	34	12,7
51-60	27	10,1
61-70	15	5,6
71-89	5	1,9
**Nivel de escolaridad**		**%**
Ninguna		1,5
Sin información		1,2
Básica primaria incompleta		10,2
Básica primaria completa		6,5
Básica secundaria incompleta		22,0
Básica secundaria completa		41,6
Técnico		6,8
Tecnológico		2,2
Pregrado		5,7
Educación continuada		1,5
Posgrado		0,7
**Ocupación**		**%**
Sin información		0,4
Ninguna		7,1
Estudiantes		9,3
Amas de casa		6,7
Trabajadores de la construcción		6,7
Conductores de vehículos		6,3
Mensajeros y repartidores		5,2
Miembros de fuerzas armadas y policía		2,9
Trabajadores de la salud		1,8
Profesionales de diferentes áreas		1,5
Oficios varios		48,3
Pensionados y jubilados		3,7
**Etnia**		**%**
Sin información		12,4
Sin pertenencia étnica		79,3
Afrodescendiente/negro		8,3
Orientación sexual		%
Sin información		3,8
Heterosexual		94,7
Homosexual		1,1
Bisexual		0,4
**Factor de vulnerabilidad**		**%**
No aplica		73,7
Sin información		2,6
Consumo de sustancias psicoactivas		16,2
Presos/detenidos		2,3
Desplazamiento interurbano		1,9
Barras de fútbol		1,1
Mujer cabeza de hogar		1,1
Comunidad LGBTI		0,8
Funcionarios judiciales		0,4

En cuanto a los hechos, la mayoría de los casos se presentó en los meses de enero (14,3 %), julio (10,9 %) y abril (10, 2%), lo que coincide con la finalización de períodos de vacaciones (diciembre, semana santa y mitad de año, respectivamente), entre las 6:00 y las 10:00 p. m. (34,6 %), y entre la media noche y las primeras horas de la madrugada (20,1 %). Los hechos ocurrieron mayoritariamente en el perímetro urbano del municipio de Cali (74 %), principalmente en las comunas 13 y 15 (15,4 %), y en muchos barrios diferentes. Los contextos más frecuentes fueron la violencia interpersonal (76,3 %) y la violencia de pareja (19,0 %) (cuadro 2).

**Cuadro 2:** Datos sobre la ocurrencia de los hechos

Mes	%
Enero	14,3
Febrero	8,6
Marzo	8,3
Abril	10,2
Mayo	6,8
Junio	4,5
Julio	10,9
Agosto	3,8
Septiembre	7,5
Octubre	8,6
Noviembre	7,5
Diciembre	9,0
**Barrio**	**%**
Sin información	8,3
Fuera de Cali (perímetro rural)	17,7
Dentro de Cali (perímetro urbano)	74,0
**Comuna**	**%**
No aplica	22,2
15	7,9
13	7,5
2	6,8
6	6,4
Otras comunas	49,2
**Contexto**	**%**
Sin información	0,8
Violencia común (interpersonal)	76,3
Violencia de pareja	19,9
Violencia intrafamiliar	2,3
Conflicto armado	0,4
Violencia infantil	0,4

En el caso del agresor, en su mayoría de sexo masculino (94,4 %), algunos se identificaron como conocidos sin ningún trato (20,7 %) y otros como pareja o expareja (20,3 %), siendo los agentes y mecanismos traumáticos más usados el contundente (88,0 %) y el proyectil disparado por arma de fuego (6,4 %) (cuadro 3).

**Cuadro 3:** Datos sobre el agresor

Agresor	%
Sin información	7,1
No identificado	3,8
Conocido sin ningún trato	20,7
Pareja o expareja	20,3
Vecino	10,9
Policía	8,6
Amigo	5,6
Otro	22,9
**Agentes y mecanismos traumáticos**	**%**
No se pude determinar	1,1
Contundente	88,0
Proyectil de arma de fuego	6,4
Corto-contundente	1,9
Cortopunzante	1,9
Sin información	0,4
Cortante	0,4

Como consecuencia de las lesiones, los componentes más afectados del sistema estomatognático fueron los tejidos blandos periorales (83,5 %) y periodontales (48,9 %) con presencia de edema (32,7 %) y heridas (22,9 %), entendidas estas como la pérdida de la solución de continuidad del tejido, además de cierta afectación de los tejidos duros de soporte, incluidos los dientes, en el 55,1 % de los casos. En una baja proporción de los casos, se comprometió el tejido óseo de los huesos de la cara, los procesos alveolares y la articulación temporo-mandibular (13,7 %). En ciertos casos se vieron involucrados los dientes (41,4 %), siendo más frecuentes la avulsión (18 %) y la subluxación (16,5 %), concomitantes con fracturas complicadas del tercio cervical (19,2 %) y no complicadas del tercio incisal (12,8 %) (cuadro 4).

**Cuadro 4:** Datos sobre lesiones en el sistema estomatognático

Tejidos y estructuras afectadas	%
Tejidos blandos	83,5
Tejidos periodontales	48,9
Labios orales	28,6
Mucosa labial	22,6
Extraoral	20,7
Articulación temporomandibular	7,9
Encías	7,5
Carrillos o bucas	4,5
Lengua	1,5
Piso de boca	1,1
Surcos mucogingivales	0,4
**Tipo de lesión en tejidos blandos**	**%**
Edema	32,7
Herida	22,9
Equimosis	9,8
Excoriación	9,4
Hematoma	5,6
Laceración	3
Avulsión	1,5
Eritema	1,1
**Tipo de lesión en dientes**	**%**
Avulsión	18
Subluxación	16,5
Concusión	13,2
Luxación	12,8
**Tipo de lesión en tejidos dentales**	**%**
Fractura complicada, tercio cervical	19,2
Fractura no complicada, tercio incisal	12,8
Fractura no complicada, tercio medio	10,2
Fractura radicular	3
Fractura complicada, tercio medio	2,3

Por último, en el caso de la incapacidad médico-legal, en el 64,7 % de los casos fue definitiva y, en el 34,2 %, provisional, con incapacidades de 18 (13,9 %) a 20 (28,6 %) días según el tipo de lesión (cuadro 5). En lo que respecta a las secuelas, se reportaron secuelas funcionales (24,1 %), estético-funcionales (22,9 %) y estéticas (11,7 %), aunque su carácter no se definió en la mitad de los casos (53 %), en tanto que, en el caso de las definidas, una baja proporción fue permanente (4,5 %) y otra, transitoria (4,1%) (cuadro 6).

**Cuadro 5:** Datos sobre incapacidad

Tipo de incapacidad	%
Definitiva	64,7
Provisional	34,2
Falta información para determinarla	1,1
**Días de incapacidad**	**%**
0	4,5
8	3,0
10	8,3
12	5,3
15	9,4
17	0,4
18	13,9
20	28,6
De 21 hasta 60	26,7

**Cuadro 6:** Datos sobre las secuelas

Tipo	%
Sin información	20,7
Sin secuelas	20,7
Funcional	24,1
Estética-funcional	22,9
Estéticas	11,7
**Carácter**	**%**
Sin información	17,7
Sin secuelas	20,7
Por definir	53,0
Permanente	4,5
Transitorio	4,1

## Discusión

El daño de componentes del sistema estomatognático como consecuencia de acciones violentas reportadas en el contexto del dictamen de lesiones personales es frecuente, con un promedio de 53,6 % casos al año entre el 2015 y el 2020. En un estudio previo en la misma Regional Suroccidente del Instituto Nacional de Medicina Legal y Ciencias Forenses, realizado entre el 2007 y el 2008, Cortés, *et al*. ^10^, encontraron un promedio anual del 35,33 % en 106 casos valorados, lo que indica un aumento en la frecuencia de casos del 18,27 % en siete años. En Bogotá, Támara-Patiño, *et al*. ^11^, reportaron que, del total de lesiones personales no fatales en individuos mayores de edad, el 76,35 % ocurrió en los contextos de violencia de pareja, violencia intrafamiliar, violencia interpersonal y violencia contra mayores de sesenta años.

Las frecuencias de ambos estudios demuestran los efectos del aumento de la violencia como fenómeno social, siendo el factor más común la intención del agresor de causar lesiones, lo que ha ocasionado una escalada en las prácticas violentas y el aumento de la gravedad de las lesiones en el sistema estomatognático. En este contexto, los profesionales de la salud bucal, dental y maxilofacial se encuentran en la primera línea cuando se trata de determinar este tipo lesiones, para lo cual resulta fundamental conocer el contexto asociado con este comportamiento, no solo desde el punto de vista clínico-asistencial, sino también, desde el médico-legal ^12^.

### Aspectos sociodemográficos

En lo concerniente al sexo de la víctima, la gran mayoría de estudios coincide en que son los hombres quienes más sufren lesiones personales a nivel general y en el sistema estomatognático. Sin embargo, Cortés, *et al*. ^10^, encontraron que el 52,6 % de los peritajes de lesiones personales se dio en mujeres, lo que, en contraste con la frecuencia hallada en el presente estudio, evidencia que desde el 2006 las lesiones personales en mujeres han aumentado en cerca del 17 %. En dicho estudio, en el contexto de la violencia de pareja, la intrafamiliar y la sexual, en el 88,1 % de los casos las víctimas fueron mujeres. Este aumento podría asociarse con las campañas orientadas a que las mujeres denuncien las situaciones de violencia en las que se ven envueltas. Además, debido a las altas tasas de feminicidio y femicidio, el dictamen se centra en la descripción de las lesiones como causa de muerte y hacia el dolo del acto violento *per se*, por lo que no se incluyen en las estadísticas de lesiones personales.

Otro aspecto relevante es la edad de la víctima. Cortés, *et al*. ^10^, reportaron que el rango de edad en el que se registraron lesiones más frecuentemente fue entre los 20 y los 29 años (49,1 %). En el presente estudio, si bien las lesiones fueron más frecuentes en individuos mayores de 18 años (77,9 %) en general, entre los 20 y los 29 años se presentó la mayor frecuencia (32,6%). Se evidencia, asimismo, una mayor participación de las mujeres, fundamentalmente entre los 15 y 24 años, en calidad de víctimas ^13^, hallazgo también reportado por Zapata-Bedoya, *et al*. ^14^.

La coherencia de estos datos obedece al rango de edad de la población de adultos jóvenes que se encuentran finalizando sus estudios y empezando su vida laboral en medio de un ambiente social que los expone a diferentes formas de confrontación, como se desprende del hecho de que la mayoría de las víctimas había finalizado la educación básica secundaria de forma completa (41,6 %) y refirió tener algún tipo ocupación (92,5 %), sobre todo en la categoría de oficios varios (48,3 %), la cual agrupa todo tipo de profesiones técnicas y operativas.

En este sentido, vale la pena mencionar que la mayoría de las víctimas procedía de municipios del Valle del Cauca. En el estudio de Cortés, *et al*. ^10^, el 83 % provenía de zonas urbanas cubiertas por la Regional Suroccidental del Instituto, cuya sede principal se encuentra en Cali. Asimismo, la mayoría de las víctimas no se reconoció como integrante de ninguna etnia, salvo el 8,3 % de individuos que se reconocieron como afrodescendientes o negros. Aunque el vacío de información en cuanto a la etnia se registró en pocos casos (12,4 %), el hecho de que un importante porcentaje (79,3 %) se haya declarado sin pertenencia étnica permite inferir lo problemática que ha resultado esta categoría sociodemográfica cuando se pregunta sobre la pertenencia étnica según los parámetros establecidos en el censo poblacional del 2005 ^15^^,^^16^.

Por último, el 94,7 % de las víctimas se presentó como heterosexual y el 73,7 % no reportó ningún factor de vulnerabilidad en el momento de los hechos; sin embargo, llama la atención que el 16,2 % se encontraba bajo los efectos de sustancias psicoactivas. En este sentido, en un análisis de la Oficina de Análisis de Información y Estudios Estratégicos de la Secretaría de Seguridad, Convivencia y Acceso a la Justicia de Bogotá, se había encontrado una relación estadísticamente significativa entre la presencia de lesiones personales y la asistencia a bares y discotecas ^8^, lo que se asocia con el consumo de alcohol, sustancia que afecta el juicio de las personas y las puede inducir a cometer actos violentos ^17^.

### Características de los hechos

En este estudio, resultó evidente que los hechos en los que se produjeron lesiones personales a causa de actos violentos ocurrieron en épocas del año específicas: las lesiones reportadas en enero se asocian con el período de vacaciones de diciembre y las fiestas de fin de año; las reportadas en abril, con la semana santa, y las reportadas en julio, con el periodo de vacaciones de mitad de año, en tanto que el inicio de la noche y el de la madrugada fueron los momentos críticos ^8^. Este hallazgo coincide con lo reportado en la literatura en cuanto al consumo de alcohol y la generación de violencia. Según el Centro de Referencia Nacional sobre Violencia, la medida en que el consumo de alcohol u otras sustancias psicoactivas media en las interacciones violentas determina las lesiones, cuya aparición viene precedida de una agresividad acumulada que se desborda durante discusiones para liberar tensiones. El alcohol es uno de los principales factores detonantes del uso de la violencia y causa de lesiones personales en el contexto sociocultural en que se dan estos hechos ^13^.

Asimismo, en concordancia con lo consignado por Cortés, *et al*., en su estudio ^10^, el 83 % de las lesiones ocurrió en zonas urbanas, dentro del perímetro urbano del municipio de Cali (74 %), principalmente en las comunas 6, 13 y 15 (21,8 %), ubicadas en la región nororiental y que registran una mayor concentración de violencia asociada con diversas variables socioeconómicas que propician comportamientos violentos como la gran densidad poblacional, la escasa escolaridad, la falta de cobertura de vigilancia, la presencia de grupos al margen de la ley y la falta de atención social gubernamental. Esto se ha explicado a partir de fenómenos de disfunción individual, familiar, comunitaria y “societal” que privilegian relaciones sociales y culturales basadas en el patriarcado, el racismo y el estigma, y que posibilitan la aparición de la violencia ^18^. Es importante resaltar que la comuna 2 concentra una gran cantidad de restaurantes, bares y discotecas con un importante consumo de alcohol.

Se sabe que los hechos que desembocaron en lesiones personales se inscriben en un contexto sociocultural en el que se jerarquiza la violencia. Cortés, *et al*. ^10^, señalaron que el 99 % de las lesiones descritas en su estudio fueron ocasionadas por violencia: el 44,3 % por violencia común y el 54,7 % por violencia intrafamiliar. En el presente estudio, la violencia común (76,3 %) y la violencia de pareja (19,9 %) fueron los contextos en los que más lesiones personales se produjeron. Según el Centro de Referencia Nacional sobre Violencia, ambos tipos de violencia tienen origen en discusiones y riñas que son responsables del 75 % de las lesiones personales, y producto de la intolerancia cotidiana y rutinaria frente al otro ^13^.

Zapata-Bedoya, *et al*. ^14^, reportaron en un contexto de violencia común, que los empleados, obreros y mensajeros fueron los más afectados, en tanto que en el contexto de la violencia intrafamiliar, las mujeres amas de casa fueron las principales víctimas.

### Sobre el agresor

Se pudo establecer que la mayoría de los agresores eran del sexo masculino (94,4 %), que conocían a la víctima, pero no tenían trato con ella, o eran pareja o expareja, o eran vecinos; así, los conflictos son más frecuentes e intensos entre personas que sostienen relaciones cotidianas y constantes que entre aquellos que solo sostienen relaciones esporádicas y transitorias ^8^.

En este sentido, Zapata-Bedoya, *et al*. ^14^, señalaron que, en un contexto de violencia común, el principal agresor suele ser un vecino o conocido, en tanto que, en el contexto de la violencia intrafamiliar, era el cónyuge o compañero. A este respecto, el Centro de Referencia Nacional sobre Violencia ha señalado que el 70 % de las lesiones personales son generadas por personas conocidas, es decir, el conocimiento previo entre el agresor y la víctima permite inferir que la espontaneidad en el uso de la violencia se asocia tanto a la intolerancia rutinaria como a conflictos específicos ^13^.

Esta espontaneidad y arbitrariedad en las relaciones sociales cotidianas, se expresan en discusiones y riñas en la vía pública y en el lugar de residencia, lo que evidencia que están circunscritas al conocimiento del agresor y a la concentración geográfica de los casos reportados, y que la relación previa entre el agresor y la víctima determina que la lesión personal sea el producto de una expresión de intolerancia y consecuencia de las diversas formas y medios de manifestación de la agresividad acumulada y contenida, la cual se desborda en una situación concreta ^12^; a veces, es mediada por el consumo de alcohol y otros factores desencadenantes que involucran diferentes agentes y mecanismos traumáticos, siendo los más usados el contundente (88,0 %) y el proyectil disparado por arma de fuego (6,4 %). Cortés, *et al*. ^10^, habían manifestado que el mecanismo causal más frecuente era el mecánico (98,1 %), siendo los principales medios el contundente (81,1 %) y el corto-contundente (10,4 %), así como las armas de fuego (0,9 %), lo que también concuerda con lo hallado por Zapata-Bedoya, *et al*. ^14^, sobre el uso más frecuente del mecanismo contundente (63 %).

### Afectación del sistema estomatognático

En el dictamen de lesiones personales, los peritos odontólogos y médicos deben hacer una completa descripción de las lesiones que involucran cualquier componente del sistema estomatognático para determinar la naturaleza de las lesiones, describirlas y ubicarlas, establecer el elemento causal, los criterios de incapacidad médico-legal en días y las secuelas.

Para facilitar la descripción, los componentes se subdividen en tejidos blandos, tejidos dentales, tejidos periodontales y tejidos duros. En el estudio, se encontró que los tejidos blandos periorales más afectados fueron los labios orales (29,6 %), la mucosa labial (22,6 %), los tejidos extraorales (20,7 %), los carrillos (4,5 %) y la lengua (1,5 %), con signos clínicos de edema (32,7 %), laceración o herida contusa (22,9 %) y equimosis (9,8 %). Por el contrario, Cortés, *et al*., encontraron que las lesiones más frecuentes provocaron equimosis (67,9 %), edema (63,2 %) y laceraciones (37,7 %), todas ellas asociadas con mecanismos contundentes que, en el contexto de la violencia común y de la violencia familiar, suelen tener como blanco el cuello, la cara y la cabeza de la víctima ^19^.

En cuanto a los tejidos duros, Cortés, *et al*. ^10^, encontraron que el 3,8 % de las lesiones involucró el tejido óseo de soporte, específicamente la fractura del maxilar superior (1,9 %) y de la mandíbula (1,9 %). En el presente estudio, el 13 % de las lesiones comprometía el tejido óseo de los huesos de la cara, los procesos alveolares y la articulación temporo-mandibular. El análisis morfofuncional del trauma de tejidos duros, considerados los huesos y los dientes, representa un componente clave en la investigación de la clínica odontológica forense y contribuye de manera decisiva al dictamen de lesiones personales en lo que respecta a la incapacidad y las secuelas ^20^.

En el caso de los dientes, Cortés, *et al*. ^10^, encontraron el 23,6 % de fracturas no complicadas de corona, el 8,5 % de fracturas complicadas de corona y el 0,9% de fracturas corono-radiculares no complicadas, bastante similar a lo hallado en nuestro estudio, en el que los dientes se vieron afectados en el 41,1 % de los casos, incluidas fracturas complicadas del tercio cervical (19,2 %), no complicadas del tercio incisal (12,8 %) y no complicadas del tercio medio (10,2 %). Estas lesiones dentales fueron concomitantes con lesiones de los tejidos periodontales. Cortés, *et al*. ^10^, reportaron, por su parte, que los tejidos de soporte dental se vieron afectados en el 66 % de los casos, con el 17,9 % de fracturas alveolares, el 17 % de luxaciones laterales y el 16 % de avulsiones dentales. En el presente estudio, en el 48,9 % de los casos hubo compromiso de los tejidos periodontales, principalmente con avulsiones dentales (18 %), subluxaciones (16,5 %), concusiones (13,2 %), luxaciones laterales (12,8 %) y lesiones de mucosa gingival de encías (7,5 %) y de surcos mucogingivales (0,4 %).

### Incapacidad médico-legal

Según la Guía Práctica para el Examen Odontológico Forense, versión 03 ^2^, esta corresponde al tiempo requerido para que se repare la alteración orgánica o fisiopatológica ocasionada, es decir, el tiempo expresado en días en el que determinada lesión logra la reparación biológica primaria. Las lesiones personales que cumplen esta condición implican secuelas debidas a la alteración morfológica, funcional, carencial u obstétrica por: 1) deformidad física transitoria o permanente; 2) deformidad física que afecta el rostro; 3) perturbación funcional transitoria o permanente de un miembro u órgano; 4) disminución funcional significativa de un miembro u órgano; 5) perturbación psíquica transitoria o permanente; 6) parto o aborto preterintencional, y 7) lesiones culposas contra el feto (3,5). Es por ello que una incapacidad médico-legal debe ser acorde a las condiciones reales de los procesos de reparación de las lesiones y ajustarse a la evolución fisiopatológica, clínica e imagenológica de las estructuras afectadas ^11^.

En el caso de lesiones personales que involucran componentes del sistema estomatognático, la gravedad y la estimación del tiempo de reparación determinan el carácter médico-legal de la incapacidad, que puede ser provisional o definitiva; de allí que el criterio clínico de los peritos odontólogos y médicos se oriente a fines jurídicos ^2^^,^^4^, pues, para tasar la pena, el juzgador debe tener en cuenta solo la incapacidad médico-legal definitiva, ya que esta le permite entender la gravedad de la lesión.

En su estudio, Cortés, *et al*. ^10^, encontraron que los días de incapacidad médico-legal provisional oscilaron entre los 10 y los 20 días, siendo más frecuente la de 15 días, con el 50 % de concordancia con el pronóstico teórico hecho cuando las lesiones aún se encontraban en evolución y se desconocía el estado final de la reparación. En el presente estudio, el 34,2 % de las incapacidades fue provisional y se estableció en 18 (13,9 %) o 20 (28,6 %) días según el tipo de lesión. Por otro lado, Cortés, *et al*., encontraron que los días de incapacidad médico-legal definitivos oscilaron entre 3 y 25 días, siendo la de 15 días (41,8 %) la incapacidad definitiva más frecuente. En el presente estudio, el 64,7 % de las incapacidades fueron definitivas. Solo en el 1,1 % no se pudo determinar la incapacidad por falta de información para determinarla.

### Secuelas morfofuncionales

Las secuelas en el contexto médico-legal corresponden a las alteraciones orgánicas, funcionales o psíquicas que afectan de manera considerable la forma o la función de una estructura anatómica o un órgano, y que pueden persistir más allá del tiempo esperado (más allá de la incapacidad médico- legal) para lograr su reparación ^2^^,^^4^.

Las secuelas más frecuentes en el sistema estomatognático son las siguientes de tipo funcional, estético o estético-funcional: 1) deformidad física que afecta el rostro ocasionada por la pérdida o fractura de un diente del sector anterior, la pérdida o fractura de varios dientes cuando no hay alguna condición previa, o sea, hay presanidad, las fracturas óseas con pérdida de tejido que producen una alteración estética importante y la pérdida de tejido en los labios o asimetría por mala cicatrización o cicatrices de carácter ostensible; y 2) perturbación funcional de los órganos de la masticación, fonación, del gusto y deglución ocasionada por la pérdida de dos o más piezas dentales consecutivas, múltiples fracturas cuspídeas, fracturas de maxilares de mal pronóstico oclusal, traumas en la articulación temporomandibular que causen mala oclusión o limitación de los movimientos mandibulares, pérdida de dientes, fracturas en el paladar, y lesiones en el velo del paladar, la lengua o los carrillos ^3^^,^^5^.

Cortés, *et al*. (10), encontraron secuelas en el 17,9 % de los casos de su estudio, quedando por definir el 4,7 %. En el presente estudio, se encontraron secuelas funcionales (24,1 %), estético-funcionales (22,9 %) y estéticas (11,7 %) que, según su carácter, se definieron como permanentes en el 4,5 % y transitorias en el 4,1 %, en tanto que un 53 % se encontraban por definir. Vale anotar que en el 20,2 % de los casos no se reportaron secuelas de ningún tipo.

En conclusión, puede plantearse que la violencia surge como expresión del desajuste entre los patrones sociales y culturales que permean y definen las relaciones, roles y situaciones en que se desenvuelven los agresores y las víctimas. Dada la heterogeneidad de las causas y de los factores asociados, la caracterización de las lesiones personales producto de actos violentos resulta fundamental para los profesionales de la salud que deben evaluar dichas lesiones, pues son ellos quienes deben atender en primera instancia a las personas afectadas por lesión en el sistema estomatognático, brindarles el tratamiento requerido, elaborar una historia clínica completa que describa de manera detallada los hallazgos y la naturaleza de la lesión, valorar la presanidad y la antigüedad de la lesión, ordenar la realización de exámenes paraclínicos a los que haya lugar y orientar la conducta adecuada con respecto al tratamiento de acuerdo con el contexto del caso.

Además, la herramienta SICLICO del Instituto Nacional de Medicina Legal y Ciencias Forenses es de gran utilidad para la investigación sobre los índices de violencia por lesiones personales que controla el sesgo de información al evitar los subregistros.

En este estudio, se pudo establecer que las lesiones personales que afectan al sistema estomatognático se dieron con mayor frecuencia en los contextos de violencia común y violencia intrafamiliar, en el cual las víctimas fueron en su mayoría mujeres y, los agresores, hombres, y que los hechos ocurrieron en sectores perimetrales urbanos y rurales de Cali con gran incidencia de casos.

En cuanto a las lesiones, los tejidos periodontales y los dientes fueron las estructuras más afectadas por agentes y mecanismos contundentes que ocasionaron avulsiones y subluxaciones dentales, edemas y heridas, las cuales derivaron en incapacidades médico-legales considerables (entre 15 y 21 días), y dejaron secuelas funcionales y estéticas principalmente en mujeres y hombres en edad productiva (entre 20 y 29 años).

Teniendo en cuenta los resultados de estudios previos, puede concluirse que se ha incrementado la frecuencia de lesiones personales en el sistema estomatognático producto de actos violentos, por lo que los profesionales de la salud bucal, dental y maxilofacial deben estar atentos para ofrecer el tratamiento odontológico indicado y orientar sobre la ruta de atención integral en casos de violencia como deber ciudadano y profesional
